# Translation into Spanish of: “Changes to publication requirements made at the XVIII International Botanical Congress in Melbourne - what does e-publication mean for you?”. Translated by Carmen Ulloa Ulloa, Lourdes Rico Arce, and Renée H. Fortunato Cambios a los requisitos para publicar realizados en el XVIII Congreso Internacional de Botánica en Melbourne - ¿qué significado tiene la publicación electrónica para usted?

**DOI:** 10.3897/phytokeys.6.1990

**Published:** 2011-09-14

**Authors:** Sandra Knapp, John McNeill, Nicholas J. Turland

**Affiliations:** 1 Department of Botany, The Natural History Museum, Cromwell Road, London SW7 5BD, UK The Natural History Museum London United Kingdom; 2 Royal Botanic Garden, Edinburgh, 20A Inverleith Row, Edinburgh EH3 5LR, UK Royal Botanic Garden Edinburgh United Kingdom; 3 Missouri Botanical Garden, PO Box 299, St Louis, MO 63166-0299, USA Missouri Botanical Garden St. Louis United States of America

## Abstract

Los cambios al *Código Internacional de Nomenclatura Botánica* se deciden cada seis años en las Secciones de Nomenclatura asociadas a los Congresos Internacionales de Botánica. El XVIII Congreso Internacional de Botánica se llevó a cabo en Melbourne, Australia; la Sección de Nomenclatura se efectuó desde el 18 al 22 de julio de 2011 y sus decisiones fueron aceptadas por el Congreso en la asamblea plenaria del 30 de julio. Como resultado de esta reunión se hicieron varios cambios importantes al *Código* que afectarán la publicación de nuevos nombres. Dos de estos cambios entrarán en vigencia a partir del 1 de enero de 2012, meses antes de la edición del *Código de Melbourne*. El material electrónico publicado en línea en Formato de Documento Portátil (PDF) con un Número Internacional Normalizado de Publicaciones Seriadas (ISSN) o con un Número Internacional Normalizado de Libro (ISBN) constituirá una publicación efectiva; y el requisito para una descripción o diagnosis en latín para los nombres de nuevos taxones será cambiado a un requisito de una descripción o diagnosis en los idiomas latín o inglés. Asimismo, a partir del 1 de enero de 2013, los nuevos nombres de organismos tratados como hongos, para ser publicados en forma válida, deberán incluir en el protólogo (todo lo asociado con el nombre en su publicación válida) la cita de un identificador provisto por un repositorio reconocido (como MycoBank). Se presenta un texto borrador de los nuevos artículos que tratan con publicación electrónica y se provee las mejores prácticas descritas.

Para incentivar la difusión de los cambios realizados en el *Código Internacional de Nomenclatura para algas, hongos y plantas*, este artículo se publicará en: *BMC Evolutionary Biology, Botanical Journal of the Linnean Society, Brittonia, Cladistics, MycoKeys, Mycotaxon, New Phytologist, North American Fungi, Novon, Opuscula Philolichenum, PhytoKeys, Phytoneuron, Phytotaxa, Plant Diversity and Resources, Systematic Botany* y *Taxon*.

## Introducción

En julio de 2011, durante el XVIII Congreso Internacional de Botánica en Melbourne, Australia, se hicieron dos cambios importantes en el *Código Internacional de Nomenclatura Botánica* (ahora llamado el *Código Internacional de Nomenclatura para algas, hongos y plantas*) que entrarán en vigencia a partir del 1 de enero de 2012. Estos cambios afectarán a todos los autores que publican nombres regidos por este *Código*. Como el *Código de Melbourne* no será editado hasta aproximadamente mediados del 2012, se ha pensado que sería útil actualizar estos cambios, particularmente aquellos relacionados a la publicación efectiva por medios electrónicos (artículos 29, 30 y 31). Para un informe conciso de todos los cambios al *Código* aceptados en Melbourne, ver McNeill et al. (2011).

Un borrador revisado del texto de los Artículos, Notas y Recomendaciones sobre publicación efectiva se proporciona a los editores y comités editoriales como ayuda en el establecimiento de las mejores prácticas descritas para la aplicación del *Código*. También se describe lo que estos cambios *no* significan, a fin de orientar a aquellos que deseen publicar nuevos nombres y tipificaciones por medios electrónicos. Se solicita a los lectores consultar el informe del Comité Especial sobre Publicación Electrónica que acompaña los cambios propuestos previamente al XVIII Congreso (Chapman et al. 2010); en ellos se expone el razonamiento para los cambios ahora aceptados en el *Código*.
